# Unveiling the translational dynamics of lychee (*Litchi chinesis* Sonn.) in response to cold stress

**DOI:** 10.1186/s12864-024-10591-w

**Published:** 2024-07-12

**Authors:** Mingming Chen, Shuangfeng Dai, Daming Chen, Haomin Chen, Naijie Feng, Dianfeng Zheng

**Affiliations:** 1 College of Coastal Agricultural Sciences, Guangdong Ocean University, Zhanjiang, 524008 China; 2 National Saline-Tolerant Rice Technology Innovation Center, South China, Zhanjiang, 524008 China; 3 Shenzhen Institute of Guangdong Ocean University, Shenzhen, 518108 China

**Keywords:** Lychee, Ribosome profiling, Cold stress, Codon occupancy

## Abstract

Cold stress poses a significant threat to the quality and productivity of lychee (*Litchi chinensis* Sonn*.*). While previous research has extensively explored the genomic and transcriptomic responses to cold stress in lychee, the translatome has not been thoroughly investigated. This study delves into the translatomic landscape of the 'Xiangjinfeng' cultivar under both control and low-temperature conditions using RNA sequencing and ribosome profiling. We uncovered a significant divergence between the transcriptomic and translatomic responses to cold exposure. Additionally, bioinformatics analyses underscored the crucial role of codon occupancy in lychee's cold tolerance mechanisms. Our findings reveal that the modulation of translation via codon occupancy is a vital strategy to abiotic stress. Specifically, the study identifies ribosome stalling, particularly at the E site AAU codon, as a key element of the translation machinery in lychee's response to cold stress. This work enhances our understanding of the molecular dynamics of lychee's reaction to cold stress and emphasizes the essential role of translational regulation in the plant's environmental adaptability.

**Supplementary Information:**

The online version contains supplementary material available at 10.1186/s12864-024-10591-w.

## Background

Lychee (*Litchi chinensis* Sonn.) is a key subtropical fruit known for its economic value, nutritional profile, exotic flavor, and visual appeal [1–5]. However, climate change, characterized by global warming and extreme temperatures, poses significant challenges and increases the incidence of abiotic stressors. These changes have profoundly affected global crop production [2, 6, 7]. Among these challenges, cold stress is particularly detrimental and affects both the survival and flavor quality of lychee [8, 9].

Cold stress can have several detrimental effects on lychee seedlings. Prolonged exposure to low temperatures can hinder the growth and vitality of lychee seedlings, leading to stunted development and increased susceptibility to diseases [10, 11]. Extreme or prolonged cold can damage tissues, impair physiological functions, and disrupt metabolic processes in lychee seedlings, resulting in overall reduced vitality. At the molecular level, cold stress can alter gene expression in lychee, leading to the accumulation of reactive oxygen species (ROS) and oxidative damage [12]. It can also affect the stability and function of cellular membranes and proteins in lychee seedlings [13]. Insufficient cold accumulation due to unusually high winter temperatures can result in inadequate floral initiation and poor flowering in lychee, ultimately affecting fruit yield and quality [14]. Additionally, the variability in cold requirements among different lychee varieties complicates effective management [15, 16]. Balancing the right amount of cold stress is crucial, as excessive cold exposure pose significant challenges to the health and productivity of lychee seedlings [17–19]. Therefore, understanding the molecular mechanisms underlying these responses in lychee is essential for developing strategies to mitigate the negative impacts of cold stress.

Gene regulatory networks for lychee's stress responses have been studied using high-throughput sequencing and bioinformatics tools, but the molecular response to low-temperature stress remains unclear [5, 8, 20]. The lack of precise genome annotation has further hindered the study of lychee's transcriptome and translatome. Plant adaptation to stress involves complex regulation, including gene expression, post-transcriptional processes, post-translational modifications, and metabolite feedback mechanisms [7, 9, 20–22]. Ribosome profiling, a high-resolution deep-sequencing technique, is crucial for analyzing RNA translation dynamics in lychee (*Litchi chinensis*) [23–26]. This method involves quantifies ribosome-protected mRNA fragments (RPFs) after RNase treatment, allowing detailed analysis of translation. Ribosome profiling has revealed shifts in translation dynamics under low-temperature stress, providing insights into ribosome coverage, translation efficiency, and codon occupancy [23–36]. High-quality data exhibit distinct 3-nt periodicity, essential for confirming the accurate translation measurement [23–35, 37, 38]. Applying ribosome profiling to lychee enables detailed investigation of translational mechanisms contributing to stress resilience. Comprehensive sequencing of the lychee genome provides an opportunity to study lychee's response to cold stress with precision [39]. Utilizing this genomic resource, our study employs ribosome profiling and RNA-seq technologies to survey the translational landscape of lychee. The methodologies advance our understanding of stress responses in higher plants and highlight the critical role of translational regulation in lychee's adaptation to a changing climate. This study, supported by the recent lychee genome sequence, allows for an in-depth investigation of lychee's response to low-temperature stress [39]. Leveraging this genomic blueprint, we undertake research to scrutinize lychee’s translational landscape, extending our understanding of stress responses and emphasizing the pivotal role of translational regulation in lychee's adaption to shifting climatic conditions.

In summary, lychee faces escalating challenges from climate change, with cold stress being a significant threat. By exploring lychee's translatome, we aim to understand its response to cold stress, offering valuable insights for crop protection and enhancement. This study underscores the importance of using ribosome profiling and translational regulation in understanding lychee's adaptation to a changing environment.

## Results

### Library preparation and assessment of ribosome-protected footprints in lychee leaves

**Fig. 1:**
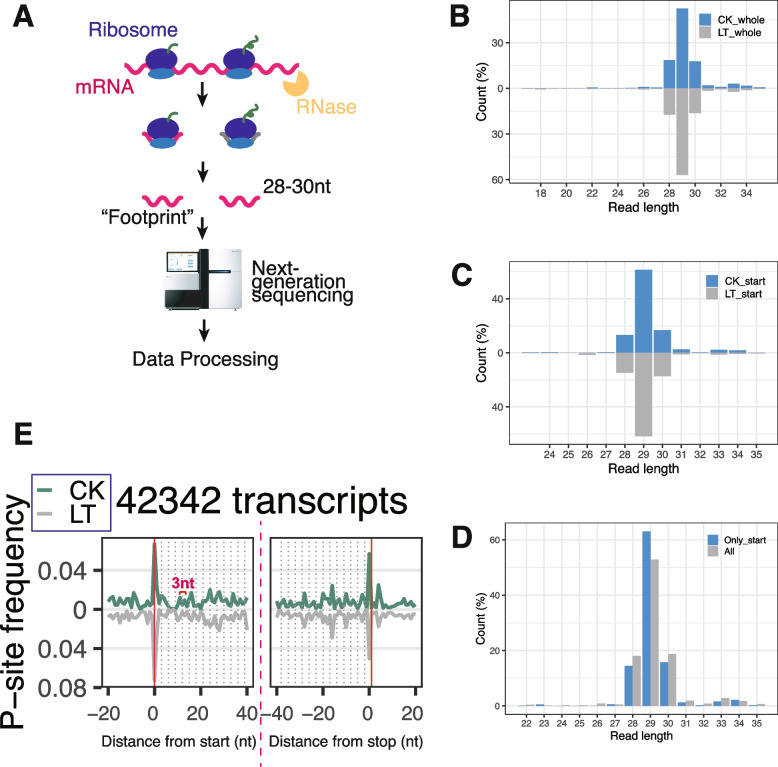
Evaluation of ribosome profiling libraries from lychee leaves. **A** Schematic representation of the ribosome profiling procedure. **B** Distribution of ribosome protected footprint lengths across the entire sequencing dataset. **C** Footprint length distribution near the start codon. **D** Comparative analysis of read length distribution at the start codon versus the entire transcript length. **E** Metagene plot depicting P-site frequency along the transcript

**Fig. 2:**
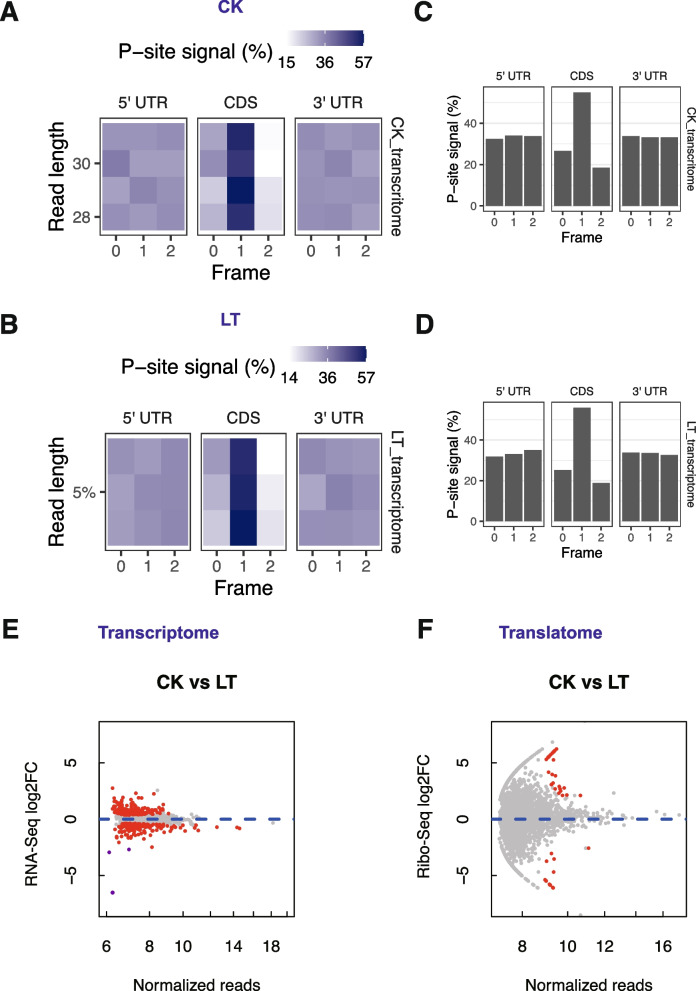
Comparison of global transcriptional and translational change. **A** P-site signal accumulation along footprint length in the regions of 5' UTR, CDS, and 3' UTR under normal conditions (CK). **B** P-site signal accumulation along footprint length in the regions of 5' UTR, CDS, and 3' UTR under cold stress (LT). **C** P-site signal distribution in the regions of 5' UTR, CDS, and 3' UTR under normal conditions (CK). **D** P-site signal distribution in the regions of 5' UTR, CDS, and 3' UTR under cold stress (LT). **E** Global changes in the transcriptome. **F** Global changes in the translatome

To elucidate the translatome landscape of *Litchi chinensis* under low-temperature stress, we conducted a comprehensive ribosome profiling study focusing on lychee leaves in the absence of low-temperature stress treatment (Fig. 1A). Employing rigorous experimental standards, we performed two replicates for each treatment condition. To identify translational differences, we employed polysome profiling (*n* = 3) to compare ribosome distribution between control and samples subjected to low-temperature stress (Fig. S1). As expected, translation was modestly suppressed under low-temperature conditions, resulting in a reduced polysome fraction (Fig. S1), confirming the temperature's impact on translation regulation. Ribosome profiling was excuted to ensure data quality and treatment efficacy, including the assessment of read lengths within the 29–31 nt range (Fig. 1B-D) and the presence of a 3-nt periodicity through metagene plots (Figs. 1E, 2A).

Preliminary processing of deep sequencing data demonstrated high reproducibility for single replicates (Fig. S1B, C). Analysis of the length distribution of ribosome-protected footprints (RPFs) in our samples revealed a predominant range of 28 to 31 nt (Fig. 1B, D). Notably, the characteristic RPF length was observed at 29 nt, indicative of monosome-protected fragments. Metagene plots of our samples also displayed periodic peaks spaced at 3-nt intervals (Fig. 1E). Previous research has confirmed that the bulk of translation footprints predominantly manifest within coding regions. However, an accumulation of queuing ribosomes is typically anticipated preceding translation initiation, along with instances of stalling near start and stop codons (Fig. 1E). This phenomenon was corroborated by the relatively heightened peaks proximate to the start and stop codons in our metagene plots. Collectively, these outcomes validate the creation of robust libraries for lychee samples, which were suitable for downstream analyses.

### Comparative analysis of lychee transcriptome and translatome landscapes under cold stress

To explore translational initiation responses across different translational features, we analyzed P-site positioning from ribosome profiling data. A strong translational signal was observed within the CDS region in both control and low-temperature groups (Fig. 2C, D), consistent with protein synthesis initiation within the coding region of RNA. Furthermore, heatmap analysis (Fig. 2A, B) showed superior periodicity of the P-site signal in the CDS compared to the 5' UTR and 3' UTR. We also compared P-site signals between control and cold stress groups. No significant differences were found, indicating that low-temperature treatment did not significantly affect global translation initiation.

In addition, we compared transcriptome fold changes using RNA-seq data with two replicates alongside ribosome profiling (Fig. S2A, B). With a significance threshold of *P*-value < 0.05, we identified genes differentially expressed at transcriptional and translational levels. Surprisingly, the transcriptome was more affected by low-temperature stress than the translatome (Fig. 2E, F**)**. RT-qPCR validation confirmed that the three most down-regulated genes (highlighted as purple in Fig. 2E) had significantly lower expression than controls (Fig. S2D). This suggests that translation dynamics are less affected by low-temperature stress in lychee. Our findings indicate a potential disparity between transcriptome and translatome responses to cold stress, warranting further investigation into regulatory mechanisms. Gene Ontology (GO) analysis on transcriptome data revealed a significant enrichment of transaltion-related pathways in samples exposed to low-temperature stress (Fig. S3A), including "mRNA cap binding complex" and "RNA cap binding complex". This suggests that cold stress broadly impacts translation processes, particularly affecting mRNA cap binding complexes. Kyoto Encyclopedia of Genes and Genomes (KEGG) pathway analysis revealed perturbations in energy supply pathways, such as the Tricarboxylic Acid (TCA) cycle (Fig. S3B), suggesting altered energy metabolism under cold stress. These insights into translation and energy metabolism pathways highlight the multifaceted molecular impact of low-temperature stress, providing a deeper understanding of the biological response to environmental challenges.

### Effects of cold stress on coding features in lychee

**Fig. 3:**
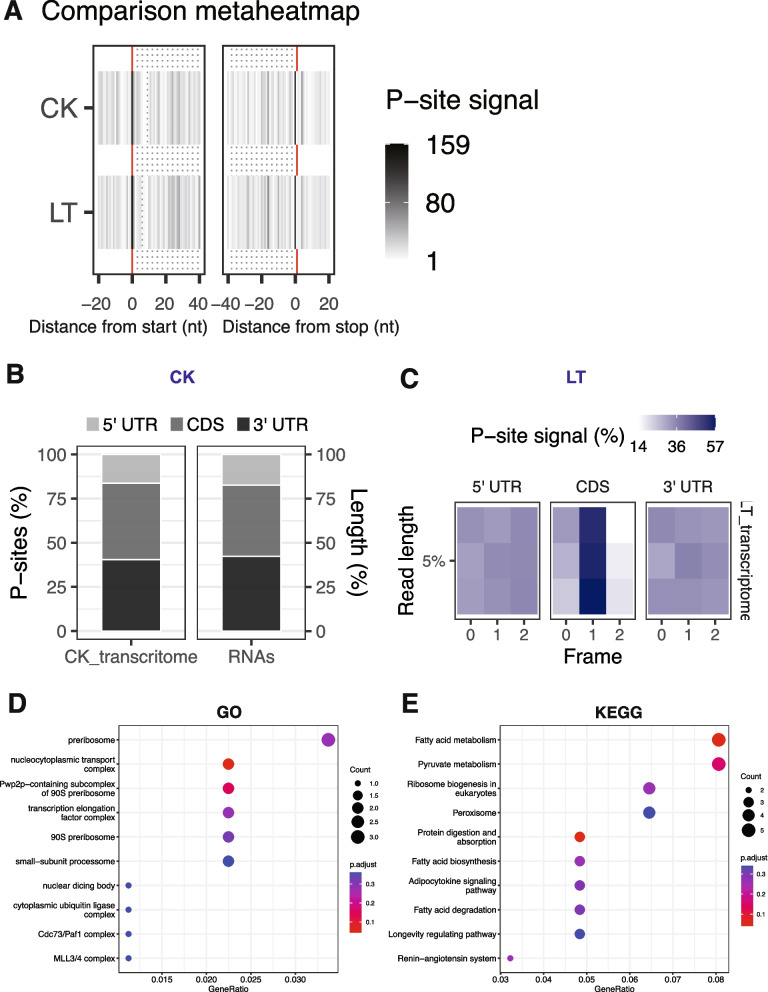
Comparison of coding features. **A** Metaheatmap of P-site signal concentrated on start and stop codon. **B** P-sites distribution along coding features evaluated from predicted model (RNAs) and transcriptome data under CK. **C** P-sites distribution along coding features evaluated from the predicted model (RNAs) and transcriptome data under low-temperature stress. **D** GO analysis for translationally affected genes under cold stress. **E** KEGG analysis for translationally affected genes under cold stress

Understanding plants’ responses to abiotic stresses through coding feature dynamics is vital for comprehending their adaptive strategies. This study focused on coding events in lychee under cold stress, a significant environmental factor. We analyzed P-site signal metaheatmaps to examine translational activity across mRNA transcripts (Fig. 3A). The metaheatmaps showed uniform signal patterns with distinct three-nucleotide periodicity, confirming the accuracy of our ribosome profiling data and providing a solid foundation for predictive modeling of translation events. Our analysis highlighted significant trends in coding localization, predominantly in the coding sequence (CDS) region, emphasizing its role in protein synthesis. In contrast, the 5' untranslated region (5' UTR) displayed the fewest coding events, suggesting it primarily contains regulatory elements. This insight underscores the complexity of translational control in *Litchi chinensis*. We also examined the independent coding distribution for each treatment, revealing a strong alignment between our prediction model and calculated values (Fig. 3B, C). This consistency underscored the robustness of our approach, Interestingly, no significant alterations in coding features were observed under of cold stress, which indicated the remarkable resilience and adaptability of lychee to environmental challenges.

Although no significant coding changes were found under cold stress, we thoroughly examined translation efficiency for potential genes and pathways. that may have been affected. Unlike transcriptome, only a few genes were impacted at the translational level (Fig. S2C). Gene Ontology (GO) and Kyoto Encyclopedia of Genes and Genomes (KEGG) analyses (Fig. 3D, E) revealed significant perturbations in ribosome function under low-temperature stress, particularly in substructures like the 'peribosome', '90S peribosome', and 'nuclear dicing body'. This raises questions about how low-temperature stress regulates ribosomes, possibly due to ribosome stalling on specific codons. In summary, our examination of coding features in lychee under low-temperature stress highlights the plant's translational responses and adaptive strategies. The consistency of our findings and the absence of significant coding events alterations underscore the plant's robustness and ability to withstand environmental stressors, revealing intriguing aspects of its adaptability.

### Codon usage analysis in *Litchi chinesis* coding sequences

**Fig. 4:**
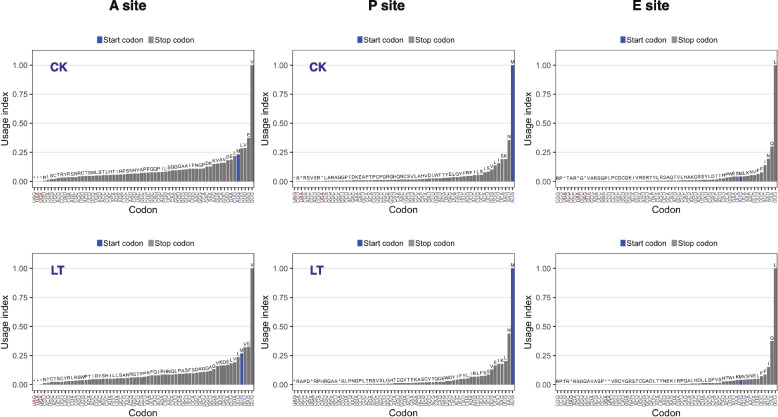
Codon usage in A, P, E Sites. Start codons are highlighted in blue, and stop codons are marked with an asterisk

We investigated codon utilization patterns using ribosome profiling data to understand how cold stress influences codon usage. Ribosome-protected reads were aligned to the lychee coding sequences, allowing direct comparisons of codon usage preferences. The diversity in codon usage across detected codons was standardized by considering the total number of codons, leading to the calculation of a codon index. A ranking plot was generated to provide a comprehensive overview of codon utilization (Fig. 4). Our analysis unveiled intriguing findings. The codon GUG (coding for Valine), the initiation codon AUG, and UUG (coding for Leucine) predominantly occupied the A, P, and E sites of the ribosome, respectively. In contrast, the termination codons UAG, UAA, and UGA exhibited relatively lower occupancy levels.

Notably, this codon preference remained consistent irrespective of low-temperature treatment, suggesting it as an inherent characteristic of lychee translation unaltered by external factors. However, some differences in codon usage were observed between the control (CK) and low-temperature (LT) treatment groups. For example, the start codon AUG occupied moved from the fifth position in the A site of the control group to the fourth position in the low-temperature treatment group.

### Cold stress facilitates AAU readthrough at E site of *Litchi chinesis* ribosome

**Fig. 5:**
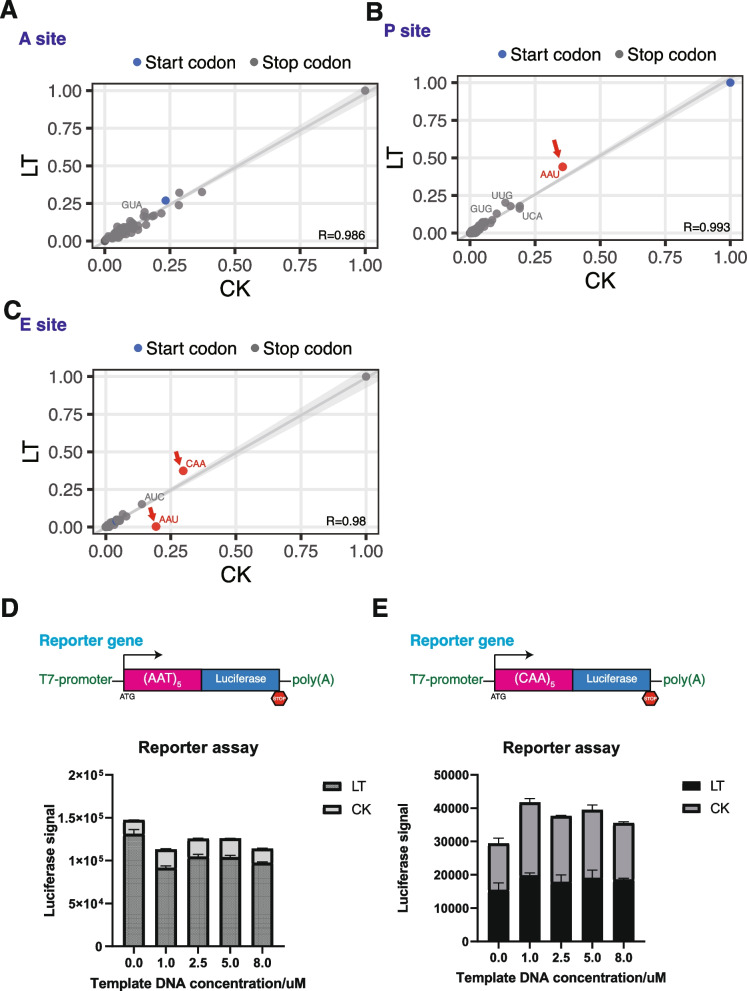
Correlation analysis of codon occupancy in A-site (**A**), P-site (**B**), and E-site (**C**). Reporter assay for reporter genes containing (AAT)_5_ (**D**) and (CAA)_5_ (**E**)

**Fig. 6:**
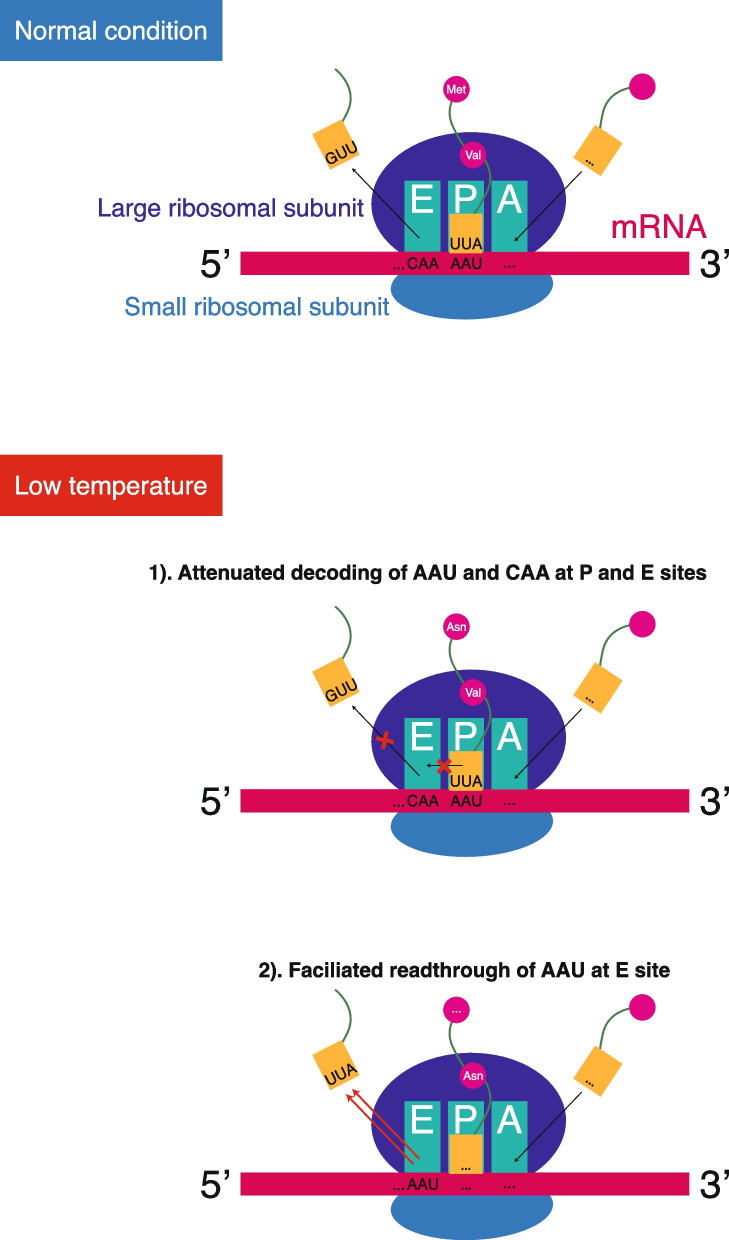
Proposed model of translational regulation under low-temperature stress in lychee

We investigated variations in codon frequency across the E, P, and A sites of ribosomes under CK and cold stress conditions. Correlation analysis revealed high coefficients of 0.986 for the A site, 0.993 for the P site, and 0.98 for the E site. The correlation plot showed similar ribosome occupancy patterns at the A sites (Fig. 5A), while distinct profiles emerged for the P (Fig. 5B) and E sites (Fig. 5C). The analysis of codon usage under low-temperature stress revealed a notable enrichment of the AAU (Asparagine) codon within the E site of the control group, with reduced occupancy of AAU and CAA (Glutamine) codons at the P and E sites. This suggested that low temperature facilitates AAU readthrough at the E site while reducing the decoding of AAU and CAA codons at the P and E sites (Fig. 6).

To validate this finding, we conducted a reporter assay using lysates from previous harvests as the source of translation factors. Lysates incubated with reporter genes containing AAU repeats (5x), showed a pronounced luciferase signal in lysates from low-temperature conditions, consistent with our codon occupancy data (Fig. 5D). This indicates that low-temperature stress enhances AAU readthrough, resulting in more robust translation. The incubation period was limited to five minutes to mitigate freeze–thaw cycle effects. Additionally, when performing the reporter assay for CAA, we observed no significant change, indicating that the readthrough of AAU on the E site is predominantly orchestrated by the primary translational machinery (Fig. 5E). This suggests that different mechanisms might be influencing codon occupancy at the P site.

## Discussion

Lychee, a tropical fruit known for its distinct flavor, is commercially grown in over twenty countries. Its productivity is limited by vulnerability to low temperatures [5, 40], posing a challenge to agricultural improvement. The lack of genomic sequencing has hindered understanding the molecular response to cold stress. The advent of the lychee genome sequence allows us to study these stress adaptation mechanisms [39]. We used deep sequencing of the lychee translatome to explore responses to cold stress. We found conserved translational dynamics in coding feature distribution, initiation site selection, and codon usage frequency. These changes suggest a conserved molecular response to environmental stress. Comparative analysis showed a significant transcriptional response to low temperatures, with 170 genes upregulated and 180 genes downregulated in the transcriptome, with 28 genes upregulated and 13 downregulated. This indicates a rapid transcriptional response, with some upregulated genes downregulated at the translational level, possibly due to mRNAs sequestration by stalled ribosomes [21, 41, 42].

Our ribosome profiling data reveal biased at the P and E sites, suggesting certain may facilitate or impede translation under cold stress [43]. This suggests lychee may adapt its translational response to environmental changes. While translation initiation is a key regulatory step, our findings and other evidence highlight the roles of ribosome stalling and elongation in responding to cold stress [24, 26, 29, 30, 35, 36]. We found no significant differences in translation initiation events between treatments, suggesting ribosome stalling is a major response to low-temperature stress. Specific ribosome stalling during cold stress may result from alterations in particular aminoacyl tRNAs or restricted amino acid availability. Lychee might accumulate specific codons during cold stresses to support stress tolerance. This aligns with previous research indicating plants often accumulates specific amino acids during abiotic stress. For example, proline accumulates in response to drought stress, helping maintain cell turgor and water balance [44]. Glutamate and arginine are linked to nitric oxide (NO) production, a crucial signaling molecule during stress responses [45]. Tryptophan accumulation is associated with the auxin pathway, involved in stress-induced growth modulation [46]. These amino acids assist in mitigating stress and are tried to signaling pathways that control plant growth, development, and defense.

Translation regulation is traditionally centered on initiation, but recent research emphasizes the significance of ribosome stalling and elongation [47–54]. We analyzed P-site signal distribution across treatment groups and found no significant impact on initiation events, suggesting ribosome stalling is the main response to low-temperature stress in lychee. Ribosome stalling under stress is well-documented and involved in a various cellular responses. For instance, oxidative stress causes ribosome stalling at tryptophan codons in fission yeast [35], and similar mechanisms regulate metabolism during ribotoxic stress in mice [36]. In plants, ribosome stalling is crucial for small RNA function [29] and epigenetic control of transposons [30]. Additionally, stalled ribosomes can also affect the cell cycle, particularly after genotoxic stress. This study provides new evidence that low-temperature stress in lychee leads to specific and genome-wide ribosome stalling at certain codons, highlighting an important aspect of the plant's stress response.

In this study, we utilized lychee seedlings to investigate the effects of cold stress on ribosome profiling, which provides insights into the dynamics of translation under stress conditions. The use of seedlings allows for controlled and reproducible experimental conditions, essential for the high sensitivity of ribosome profiling. Seedlings offer uniform and high-quality samples, minimizing variability and ensuring reliable data. This approach also helps us understand how early exposure to cold stress influences physiological and molecular processes that set the stage for flowering and fruit production. However, using seedlings instead of mature plants presents limitations. Seedlings do not fully replicate the complex interactions encountered by mature plants in field conditions, such as soil composition, water availability, and biotic interactions. Despite these limitations, understanding the early stress responses in seedlings is crucial, as it provides insights into optimizing conditions for better flowering and fruit production in lychee plants.

## Conclusions

In sum, our comprehensive study maps the transcriptional and translational landscape of lychee under low-temperature stress, revealing key insights into its molecular stress response (Fig. 6). These findings provide a valuable framework for future research aimed at enhancing cold tolerance in lychee, which could extend its cultivation range and boost agricultural productivity.

## Methods

### Sample collection and preparation

In our study, we focused on cultivable seedlings of the lychee (*Litchi chinensis*) cultivar ‘Xianjinfeng’. The seeds of the cultivar ‘Xianjinfeng’ were provided by the South Subtropical Crops Research Institute, Chinese Academy of Tropical Agricultural Sciences (Zhanjiang, Guangdong Province, China). These seeds were sown in a controlled environment, which involved growing the seedlings in a greenhouse with meticulously regulated conditions to ensure optimal germination and growth. The controlled environment included maintaining a constant temperature of 25 °C ± 2 °C, humidity levels between 60–70%, and a 16-h light/8-h dark photoperiod using full-spectrum grow lights to simulate natural sunlight. Humidity was controlled using automated misting systems, and the temperature was regulated with heating and cooling systems to prevent fluctuations. Soil moisture levels were monitored and adjusted using a drip irrigation system to ensure consistent hydration without waterlogging. The seedlings were grown under these conditions to promote healthy development, including regular monitoring and adjustment of environmental factors. After reaching an appropriate size, marked by the development of a robust root system and several sets of true leaves, the seedlings were transplanted to the trial site, which had been prepared to match the controlled conditions as closely as possible. Post this initial phase, half of seedlings were relocated to a chamber with a reduced temperature of 15 °C, while ensuring consistent hydration. After a dedicated cultivation span of 35 days, we meticulously harvested leaves from a consistent apical leaflet position. These leaves were then segregated randomly into two sets and promptly frozen in liquid nitrogen, ensuring the preservation of their molecular attributes. Characteristically, these lychee trees possess a semi-circular canopy with an open structure and exhibit moderate vigor. They belong to the late-ripening category, with their fruits maturing primarily in the upper to middle part of July. The soil at our experimental site presented the following nutrient profile: pH 4.85, available nitrogen 43.71 mg·kg^−1^, available phosphorus 108.81 mg·kg^−1^, available potassium 27.47 mg·kg^−1^, and organic matter 10.61 mg·kg^−1^.

### Polysome profiling

Polysome profiling was conducted following established protocols [24–26]. To summarize, the collected leaf samples were homogenized and transferred to 1 mL of ice-cold extraction buffer composed of 100 mM Tris–HCl (pH 7.5), 20 mM NaCl, 40 mM KCl, 20 mM MgCl_2_, 1 mM DTT, 100 µg/mL cycloheximide, and 10 U/mL DNase I. The supernatant containing the lysate was obtained by centrifugation at 2000 g and 4 °C for 2 min. Subsequently, polysome profiling was initiated by loading the sample onto a sucrose gradient ranging from 15 to 60% (w/v). The extracted RNAs were then subjected to high-speed centrifugation at 160,000 g using an SW-55 rotor (BECKMAN, USA) at 4℃ for 3 h. Fractionation, absorbance measurement, and data acquisition were carried out using a gradient station system (BRANDEL, USA).

### Ribosome profiling and RNA-seq

Library construction for ribosome profiling followed the manufacturer's instructions, employing the Ribosome Profiling Kit (GeneRbiotek). To deplete rRNA, the RiboRNA Depletion Kit (GeneRbiotek) was utilized. Dephosphorylation was achieved by adding T4 PNK (polynucleotide kinase) and ATP, followed by incubation at 37 °C for 30 min. RNA purification was performed using the RNA Clean & Concentrator kit (Zymo Research), and the purified RNA was used for library construction with the QIAseq miRNA Library kit (QIAGEN). Both Ribo-seq and RNA-seq libraries were subjected to sequencing on an Illumina HiSeq4000 platform using a paired-end 150-bp sequencing strategy.

### RT-qPCR

RT-qPCR was performed to quantify the expression of genes in lychee leaves from control and low-temperature (LT) treated samples. Total RNA was extracted from each sample and cDNA was synthesized from the extracted RNA. The cDNA was then used as the template for qPCR, which measures the amount of LITCHI012237.m1, LITCHI013690.m1, and LITCHI007308.m1 mRNA in each sample. The relative expression was calculated using the ^ΔΔ^Ct method [55, 56], and β-Actin was used as the housekeeping gene. Statistical analysis revealed that the expression of genes was significantly down-regulated in the LT compared to the control (*p* < 0.05). The primers used for quantifying the above 3 genes are listed as below:

**Table:** Primers for RT-qPCR

Name	Forward primer	Reverse primer
LITCHI012237.m1	5'-ATGATCTTTTGGACATCTTTGTTGTT-3'	5'-CTGTTGCTTCTGACTTGTATCTGTCCCAAAT-3'
LITCHI013690.m1	5'-ATGAAAGGAGATGGGTATGTGCC-3'	5'-GTGTTTCCTTCTCCGCTTTCTT-3'
LITCHI007308.m1	5'-ATGGGTTCAGAATTGGCAGCAA-3'	5'-CAGCACAAGGCCTGTTATGG-3'

### Reporter assay

For the in vitro translation assay, 30 µL of the crude plant lysate harvested during ribosome profiling was incubated with 0–10 µM of a luciferase reporter DNA oligo (Synthesized by IDT) in a 50 µL translation buffer, and the mixture was then incubated at 25 °C for 10 min. Luciferase activity was assessed using the Promega Luciferase Assay System (Promega, E1501). Specifically, 10 µL of the translation reaction was combined with 90 µL of the luciferase assay reagent from the kit, and the resulting luminescence was immediately quantified with a luminometer. Data were analyzed by normalizing luciferase activity against total protein concentration, as determined by the Bradford assay. All measurements were performed in triplicate, and data are presented as the mean ± standard deviation. Statistical significance was determined using a t-test, with a threshold of *p* < 0.05.

### Data processing and analysis

Raw reads from the ribosome profiling and RNA-seq experiments were aligned to the reference genome using STAR and subsequently trimmed using the fastp tool [27]. Identification of rRNA contamination utilized an rRNA reference predicted from the genome reference with HMMER2 [28]. Reads contaminated with rRNA were excluded from downstream analysis, and FPKM (Fragments Per Kilobase Million) values were quantified using RSEM [32]. Differentially expressed genes, both upregulated (log_2_(fold change) > 1, *P*-value ≤ 0.05) and downregulated (log_2_(fold change) < 1, *P*-value ≤ 0.05), were determined with the R package edgeR [34]. To gain insights into translational dynamics and codon usage, various analyses were conducted. These included assessing length distribution, generating metagene plots to illustrate 3-nt periodicity, examining codon frequency, determining ribosome stalling rates, and assessing the correlation of codon occupancy. All these analyses were performed using the R package "riboWaltz" [31]. Codon frequency and occupancy were computed by comparison with genome-wide usage.

### Experimental replicates and statistical analysis

For robust statistical analysis, biological replicates were collected and processed independently. Data are presented as means ± standard error of the mean (SEM), and statistical significance was assessed using appropriate statistical tests, as indicated in the respective analyses. The chosen significance threshold was a *p*-value of ≤ 0.05, denoting statistical significance.

### Quality control and preprocessing

Prior to downstream analysis, quality control steps were implemented to ensure the reliability of the data. This included filtering low-quality reads, assessing sequencing depth, and checking for potential batch effects or outliers. All preprocessing steps were conducted using widely accepted bioinformatics tools and scripts.

### Data visualization

The presentation of results was facilitated through the use of various data visualization techniques. Graphs, heatmaps, and other visual representations were generated using specialized bioinformatics and statistical software to provide a clear and comprehensive illustration of the findings.

### Supplementary Information

Supplementary Material 1: Fig. S1. Polysome profiling of lychee leaves under cold stress (LT) or normal condition (CK) (A). Correlation analysis for two replicates for CK (B) and LT treatments (C). Fig. S2. Correlation between replicates under CK and cold stress conditions (A and B), scatter plot of transcripts defined by translation efficiency (C), and RT-qPCR of the most downregulated genes under cold stress (D). Fig. S3. Pathway enrichments analysis. (A) GO analysis for transcriptionally affected genes under cold stress. (B) KEGG analysis for transcriptionally affected genes under cold stress.

## Data Availability

The raw sequencing data, processed datasets, and analytical scripts are accessible upon request, promoting transparency and reproducibility of our study. Additionally, all sequencing data have been deposited to SRA (Sequence Read Archive) database (Project number: PRJNA1030889, https://www.ncbi.nlm.nih.gov/bioproject/?term=PRJNA1030889; https://www.ncbi.nlm.nih.gov/sra/?term=PRJNA1030889), ensuring that they are publicly available for further investigation by the scientific community. The datasets used and/or analysed during the current study would be available from the corresponding author on reasonable request.
