# Supplementary material for: Unveiling the translational dynamics of lychee (Litchi chinesis Sonn.) in response to cold stress
Source: BMC Genomics. 2024 Jul 12;25:686. doi: 10.1186/s12864-024-10591-w (PMC11241792; doi:10.1186/s12864-024-10591-w)
Supplement: Supplementary file 1 — Supplementary Material 1: Fig. S1. Polysome profiling of lychee leaves under cold stress (LT) or normal condition (CK) (A). Correlation analysis for two replicates for CK (B) and LT treatments (C). Fig. S2. Correlation between replicates under CK and cold stress conditions (A and B), scatter plot of transcripts defined by translation efficiency (C), and RT-qPCR of the most downregulated genes under cold stress (D). Fig. S3. Pathway enrichments analysis. (A) GO analysis for transcriptionally affected genes under cold stress. (B) KEGG analysis for transcriptionally affected genes under cold stress. [file 12864_2024_10591_MOESM1_ESM.docx]

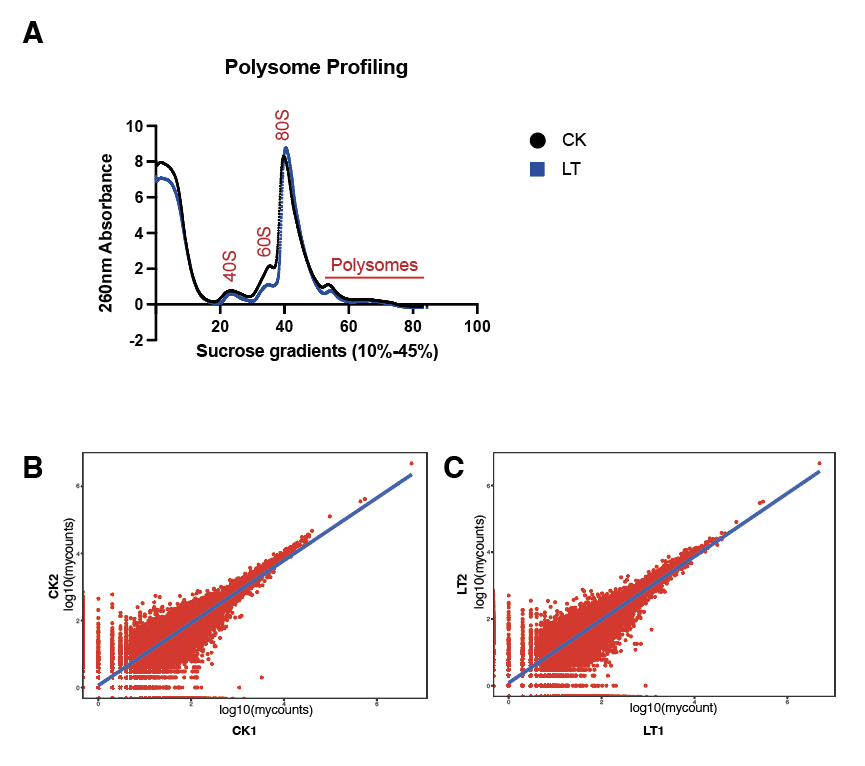


**Fig.S1 Polysome profiling of lychee leaves under cold stress (LT) or normal condition (CK) (A). Correlation analysis for two replicates for CK (B) and LT treatments (C).**


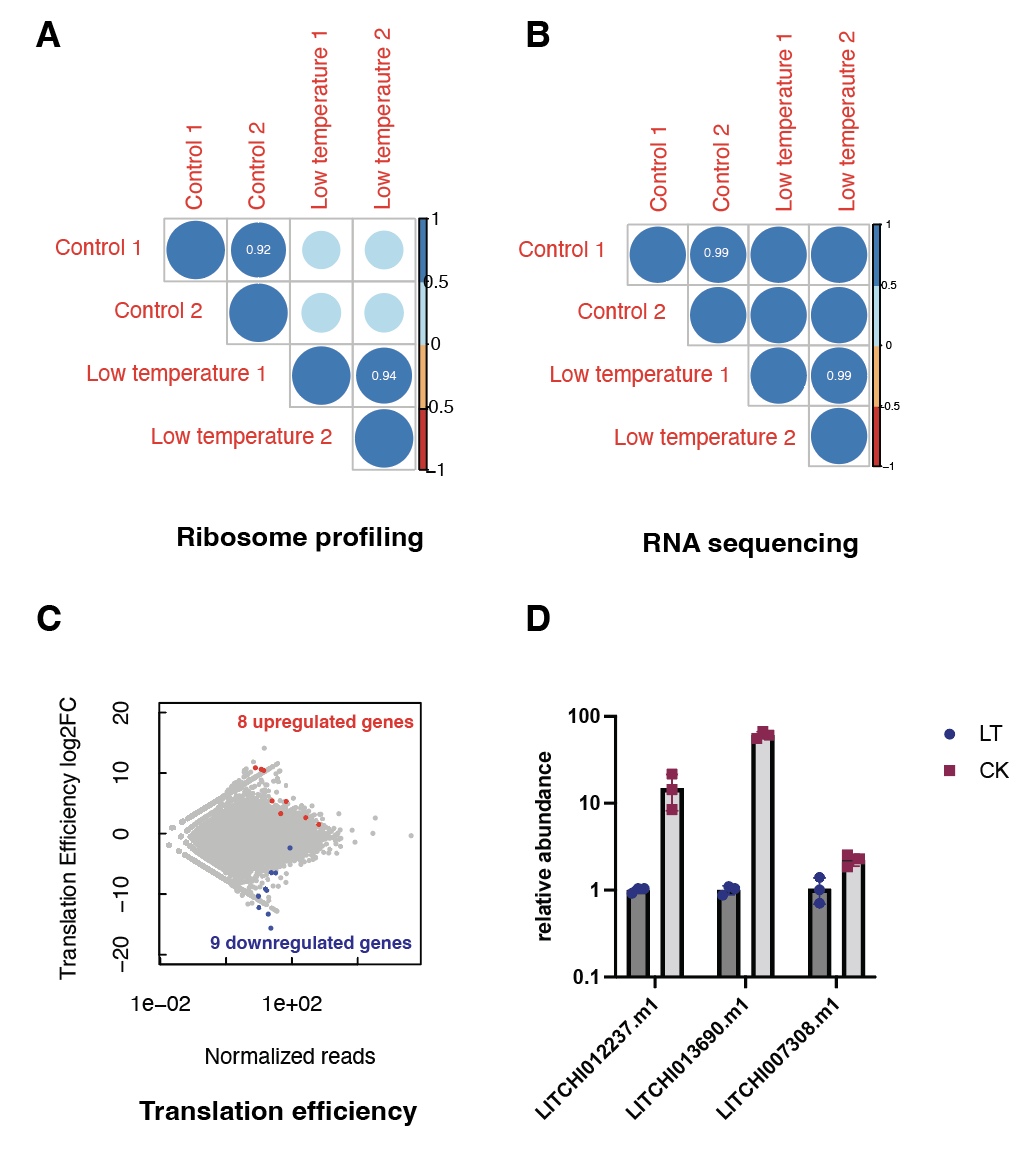


**Fig.S2 Correlation between replicates under CK and cold stress conditions (A and B), scatter plot of transcripts defined by translation efficiency (C), and RT-qPCR of the most downregulated genes under cold stress (D).**


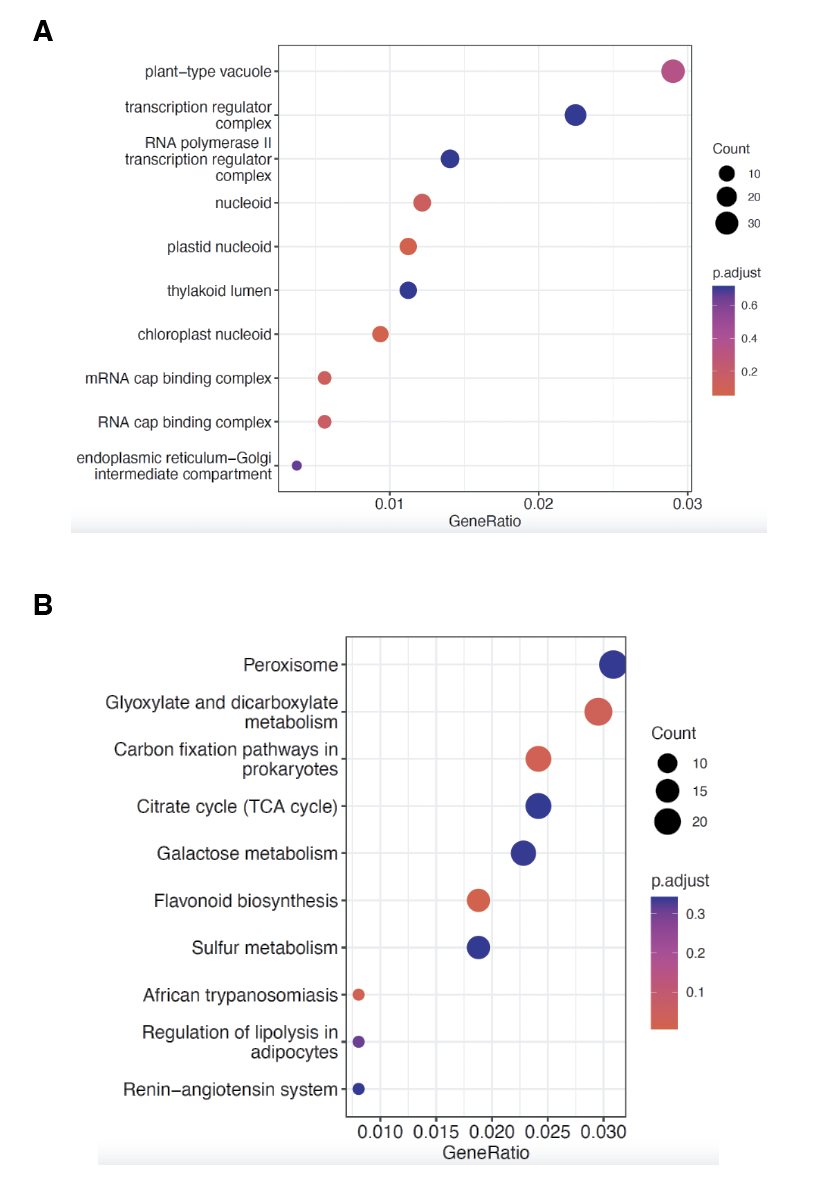


**Fig. S3 Pathway enrichments analysis. (A) GO analysis for transcriptionally affected genes under cold stress. (B) KEGG analysis for transcriptionally affected genes under cold stress.**
